# Effects of motor and cognitive dual-task performance in depressive elderly, healthy older adults, and healthy young individuals

**DOI:** 10.1590/S1980-57642011DN05030007

**Published:** 2011

**Authors:** Helena Moraes, Andrea Deslandes, Heitor Silveira, Cynthia Arcoverde, Heloisa Alve, Jerson Laks

**Affiliations:** 1 Ms, Center for Alzheimer Disease and Related Disorders, Institute of Psychiatry of the Federal University of Rio de Janeiro, Rio de Janeiro RJ, Brazil; 2 PHD, National School of Public Health, FIOCRUZ, Rio de Janeiro RJ, Brazil; 3 Ms, Beckman Institute & Department of Psychology, University of Illinois at Urbana-Champaign; 4 PHD, Researcher Pq2 from the National Research Council (CNPq)

**Keywords:** exercise, cognition, depression, aging, dual-task

## Abstract

Impairments in dual-task performance can be observed in healthy older adults when motor and cognitive assignments are applied simultaneously. According to the hypofrontality hypothesis, there may be a reduction in frontal cognitive function during exercise.

**Objective:**

The aim of the present study was to compare the performance changes on cognitive tests of depressive elderly (n=10), healthy older adults (n=10), and healthy young individuals (n=10) during cycle ergometer exercise.

**Methods:**

The groups were submitted to a working memory test, a short memory test and a semantic memory test, before and during a 20-minute cycle ergometer exercise at 80% of their age-predicted maximal heart rate.

**Results:**

Significant differences (p=0.04) were observed in scores on the digit backward test during exercise when young individuals were compared to healthy older adults. This result indicates that young subjects, as expected, had better performance than elderly. No significant differences were found among the groups for the digit forward subtest (p=0.40) or the vocabulary test (p=0.69).

**Conclusion:**

Data from this study showed that healthy older adults had impaired performance on higher cognitive tasks when these assignments were applied together with motor tasks.

## Abstract (pt)

Prejuízos no desempenho de dupla tarefa podem ser observados em idosos saudáveis quando tarefas motoras e cognitivas são aplicadas simultaneamente. De acordo com a hipótese da hipofrontalidade, durante o exercício pode ocorrer uma redução no desempenho de tarefas cognitivas frontais.

**Objetivo:**

O objetivo do presente estudo foi comparar as modificações no desempenho cognitivo durante o exercício em bicicleta ergométrica em idosos depressivos (n=10), idosos saudáveis(n=10), e jovens saudáveis (n=10).

**Métodos:**

Os grupos foram submetidos a teste de memória de trabalho, teste de memória de curto prazo e teste de memória semântica antes e durante 20 minutos de exercício em bicicleta estacionária a 80% da frequência cardíaca máxima prevista pela idade.

**Resultados:**

Foi observada uma diferença significativa nas mudanças do escore do teste *digit backward* quando comparados os grupos de jovens e idosos saudáveis (p=0,04), a qual indicou que o jovem mostrou melhora no desempenho, enquanto o grupo de idosos saudáveis declinou. Não houve diferença significativa entre grupos para o subteste de dígitos ordem direta (p=0,40) e teste de vocabulário (p=0,69).

**Conclusão:**

Os resultados da nossa investigação mostrou que pessoas idosas saudáveis apresentaram prejuízo no desempenho de tarefas cognitivas complexas quando desempenhadas durante a tarefa motora.

## Introduction

Depression in older adults is a mood disorder with multiple risk factors whose development may be associated with the environment of the elderly as well as their lifestyles.^1^ Several studies in the literature have documented that depressive elderly usually display impaired executive function and psychomotor retardation.^2-4^ These findings may be associated with the neuropsychological and anatomical changes that occur during aging, particularly in the prefrontal cortex.^5^

The prefrontal cortex plays a significant role in high-order cognitive skills, such as working memory, executive function and dual-task cognitive performance.^6^ Moreover, the prefrontal cortex may play a fundamental role during the accomplishment of motor tasks, since some cognitive parameters (such as attention) are required to perform these skills.^7^ Especially in elderly individuals, motor tasks require higher levels of control of executive processing and memory leading to a greater difficulty in executing two tasks at the same time.^8-12^ Furthermore, recent studies have observed that the frontal cortex is activated while learning a new motor task, an unfamiliar exercise or an exercise at higher intensity.^7,13^

With regard to neurophysiological findings, some theories have been postulated to explain different changes that occur during dual task performance. According to the hypofrontality hypothesis, the impairment of higher cognitive tasks during exercise may be associated with a redirection of metabolism toward the motor area, and a consequent reduction of the metabolism in the frontal area.^14^ The capacity-sharing theory suggests that one, or both, of the tasks is impaired, even when they are over-learned and largely automatic^15^ However, the bottleneck theory and the multiple resource models have proposed that decreases in performance during dual-tasks depend on the neural networks involved or the number of resources being used to perform the tasks.^9^

Several studies have found that level of cognitive performance during physical training may be associated with both intensity and duration of the exercise, whereby moderate loads induce benefits in cognition, while low and high loads promote negative results.^16^ Because normal brain and cognitive changes take place in healthy aging, and given depression in aging promotes severe impairments in cognitive functions, cognitive performance in depressive elderly during exercise may be lower. However, few studies have investigated the impact of dual tasking using both a cognitive and a motor task simultaneously in older adults.

Therefore, the purpose of the present study was to compare the performance on cognitive tests during physical exercise among depressive elderly, healthy older adults, and young individuals. We have hypothesized that the difference among the groups would be more evident in the most complex cognitive test and between healthy young and depressive elderly groups.

## Methods

The study sample consisted of 30 subjects, divided into three groups: depressive elderly (n=10, including one male), healthy older adults (n=10, including two males), and young individuals (n=10, including three males). Participants of the study were physically active for at least six months, although were not engaged in competitive sports. Healthy elderly and young subjects were selected at local fitness centers based on a detailed medical history questionnaire. Depressive subjects were diagnosed with MDD according to the DSM-IV.^17^ and had been in use of medication (antidepressants and anxiolytics) for at least six months at the time of the evaluation. Healthy young individuals aged from 20 to 30 years, as well as healthy older adults and depressive elderly older than 60 years of age were included in the study. Inclusion criteria were: previous experience on a cycle ergometer, literacy level, mini-mental state examination^18^ ≥24, healthy young individuals and healthy older adults with no physical or mental disorders, and depressive elderly with no other comorbidities.

### Experimental procedures

All sessions were conducted at the same time of day (1:00pm-5:00pm) within a controlled environment (without any external stimuli that could distract participants’ attention). The experimental procedures were approved by the Psychiatric Institute’s Ethics Committee. All participants signed a written informed consent agreement prior to participation in the study.

Before the sessions, participants were submitted to the Beck Depression Inventory (BDI)^19^ and two neuropsychological tests. Subsequently, participants performed a 20-minute exercise session on a cycle ergometer (Monark^®^) being instructed to maintain the same intensity (at 80% of their age-predicted maximal heart rate).^20^ Five minutes into the exercise session, neuropsychological tests were administered again. In order to control exercise intensity, heart rate was monitored by a telemetric monitor (Polar^®^, Brazil). The Borg Scale was applied as an auxiliary measure to control perceived effort by the participant.^21^ This scale represents a simple method of grading perceived exertion by test participants, with values ranging from 6 to 20 (the higher the perceived value of participant, the greater the effort exerted).

### Neuropsychological tests

The digit span test was administered to measure prefrontal-dependent cognition.

The Digit span is part of the WAIS-R battery^22^ and measures short-term memory, working memory, and attention. It is comprises forward and backward subtests, where subjects must repeat a series of digits in direct order (forward) and inverse order (backward). There is a progressive increase in the number of digits after each successful response. The maximum score is 14 points for both subtests.

The vocabulary test was administered as a stable test which is not associated with frontal function. Vocabulary is also a subtest of the WAIS-R^22^ and measures semantic long-term memory. The subject is asked to provide the meaning of 35 words. Responses are scored between 0 and 2, according to a predefined rating scale. The maximum score is 70.

### Statistical analyses

The SPSS software (version 16.0) was used to analyze all data. Homogeneity of variance (Levene’s test) and normality (Shapiro Wilk’s test) were tested. One-way ANOVA and Kruskall-Wallis tests were used for parametric and non-parametric data, respectively. When the difference was statistically significant (p≤0.05), pairwise comparisons were performed using the Tukey or Tamhane’s post-hoc tests. Cognitive changes during exercise were evaluated using delta values (“during” minus “pre-exercise”) among groups.

## Results

The descriptive analysis of the subjects (age, years of education and BDI) is shown in Table 1. As expected, a significant difference was observed for age (p<0.001) and BDI (p<0.001); the young group was significantly younger (p<0.001) and the depressive group had a higher BDI score compared to both healthy elderly (p=0.04) and young (p=0.01) groups. A significant difference in years of education was observed only between the depressive and young groups (p=0.006). Non-significant differences were observed for age between depressive and healthy elderly adults (p=0.80) and for BDI scores between young adults and healthy elderly (p=0.43). The Borg scale showed no differences among groups (p=0.11).

**Table 1:** Comparison of sample characteristics (values expressed as median (interquartile range 25-75%).

	Depressive elderly(DE=10)	Healthy elderly(HE=10)	Healthy young(HY=10)	p (two-tailed)	Multiple comparison
Age (years)	74 (72.2-75)	70 (66.5-73)	24 (22.5-26)	<0.001	DE≠HY HE≠HY
Years of education (y)	9.5 (8-11)	15 (11- 16.7)	15 (15-15)	0.006	DE≠HY
BDI	10.5 (4.2-11)	3 (0.25-3.75)	0 (0-2)	<0.001	DE≠HE DE≠HY

BDI: Beck Depression Inventory.

Before exercise, a significant difference among groups was seen on the digit backward subtest (p<0.001), digit forward subtest (p<0.001), and on the vocabulary test (p=0.004). Post hoc analysis indicated significant differences between young adults and healthy elderly on the digit backward and forward subtests (p=0.026; p=0.017, respectively), and between young adults and depressive elderly (p<0.001; p<0.001) on the same subtests. Moreover, for the vocabulary test, the only significant difference observed was between the young and depressive groups (p=0.004) (Table 2).

**Table 2:** Neuropsychological tests before and during exercise among depressive elderly, healthy elderly and healthy young (values expressed as mean (standard deviation)).

	Depressive elderly (DE=10)	Healthy elderly (HE=10)	Healthy young (HY=10)	p (two-tailed)	Multiple comparison
Digit backward					
Before	4.3 (1.6)*	6.1 (2.1)*	9.3 (2.7)*	<0.001	DE≠HY
During	4.1 (1.8)	5.2 (1.5)	10 (2.9)		HE≠HY
Digit forward					
Before	4.9 (1.4)*	6.3 (2.2)*	9.2 (1.9)*	0.006	DE≠HY
During	4.8 (2.8)	6.7 (0.9)	10 (2.3)		HE≠HY
Vocabulary					
Before	53.4 (9.5)*	63.5 (9.2)	66.8 (2.7)*	<0.001	DE≠HY
During	51 (10)	62.4 (8.2)	66 (3.3)		

* Difference among groups (p≤0.05).

A comparison of delta values among groups was performed. A significant difference was observed on the digit backward subtest between the young and the healthy elderly groups (p=0.04). The young group showed a positive delta, indicating an improvement on this subtest during exercise, while the healthy elderly group had a negative delta showing a decline in performance. No significant difference between groups was found for the digit forward subtest (p=0.40) or vocabulary test (p=0.69) (Table 3).

**Table 3:** Comparison of neuropsychological test deltas (during-pre-exercise) among groups (values expressed as mean (standard deviation)).

	Depressive elderlyN=10	Healthy elderly N=10	Healthy young N=10
Digit backward	-0.2 (1)	-0.9 (1.3)*	0.8 (1.6)*
Digit forward	-0.1 (2)	0.4 (2.1)	0.9 (1.4)
Vocabulary	-2.4 (4.8)	-1.1 (2.1)	-0.8 (1.8)

BDI: Beck Depression Inventory;

* Difference among groups (p≤0.05).

## DISCUSSION

The results of the present study indicated that, during dual-task performance, significant changes were evident on the digit backward test for healthy young individuals compared to healthy older adults. This finding suggests that healthy older adults are more sensitive to dual-task interference. This effect appears to be greater during attention and working memory tasks.

With regard to baseline, the depressive group showed significantly lower years of education compared to the young group. However, since the mini-mental state examination was used to equalize cognitive function across groups, this difference may not have influenced the results. Moreover, the healthy elderly group showed lower scores on the digit span subtests compared to the young group while no significant difference was found on the vocabulary test. According to Park et al. (2001)^,^^23^ several cognitive functions decline during normal aging, but vocabulary remains relatively unchanged. The non-significant results in comparisons between the depressive and healthy elderly groups for all the tests were not in accordance with the findings of previous studies reporting a positive correlation between depressive symptoms and cognitive deficits.^24^

During exercise, delta values of the digit backward subtest revealed that healthy elderly adults showed a worsening in performance while the young group showed an improvement. This test has a higher cognitive demand compared to the others and could have influenced the result. The hypofrontality hypothesis suggests that significant cognitive impairment during exercise is observed only on tasks of higher complexity.^25^ According to cognitive-energetic models, impairment in cognitive performance can occur when exercise and cognitive tasks compete for resources and effort.^26^ Moreover, according to the bottleneck theory, impairment in motor or cognitive tasks takes place if neural networks involved in the two processes overlap.^9^ Although the subjects involved in the study were adapted to regular physical activity, maintaining the intensity of exercise through a constant pedaling cadence demanded much higher levels of attention.

Previous studies have shown that cognitive impairment during exercise in young individuals is observed in exercises of longer duration and with more complex cognitive tasks.^25,27^ The findings of the present study suggest that interference of moderate exercise in cognitive performance occurs only among healthy elderly subjects. No significant differences were observed among depressive elderly and the other experimental groups. This unexpected result could be associated with lower scores on the cognitive tests at baseline, i.e. the lack of statistical differences could be due to floor effects.

Other unexpected results were observed regarding the vocabulary test results, given non-association with frontal function and aging, where negative delta values on the vocabulary test for all groups indicated worse performance during exercise. Lower scores on the vocabulary test might be caused either by increased impulsivity or decreased accuracy in defining the correct words during exercise.

Akin to several previous studies,^25,28,29^ a major limitation of the present investigation was its small sample size. The lack of more significant results on group comparisons might have been influenced by this limitation. Further studies should be conducted investigating the effects of aging on dual-task performance that include patients with possible cognitive impairment and examine the effects of physical fitness. Other cognitive tasks should also be tested.

The results of the present investigation showed that elderly individuals exhibit impairment in higher cognitive task performance during motor tasks. Enhancing knowledge about dual-tasking is of major importance, since many activities of daily living involve performance of more than one task at a time. In addition, dual-task interference is associated with a higher risk of falls in older adults.
